# A β‐mannan utilization locus in *Bacteroides ovatus* involves a GH36 α‐galactosidase active on galactomannans

**DOI:** 10.1002/1873-3468.12250

**Published:** 2016-06-28

**Authors:** Sumitha K. Reddy, Viktoria Bågenholm, Nicholas A. Pudlo, Hanene Bouraoui, Nicole M. Koropatkin, Eric C. Martens, Henrik Stålbrand

**Affiliations:** ^1^ Department of Biochemistry and Structural BiologyLund UniversitySweden; ^2^ Department of Microbiology and ImmunologyUniversity of Michigan Medical SchoolAnn ArborMIUSA

**Keywords:** *Bacteroides ovatus*, galactomannan modification, GH36 α‐galactosidase, polysaccharide utilization locus

## Abstract

The *Bacova_02091* gene in the β‐mannan utilization locus of *Bacteroides ovatus* encodes a family GH36 α‐galactosidase (BoGal36A), transcriptionally upregulated during growth on galactomannan. Characterization of recombinant BoGal36A reveals unique properties compared to other GH36 α‐galactosidases, which preferentially hydrolyse terminal α‐galactose in raffinose family oligosaccharides. BoGal36A prefers hydrolysing internal galactose substitutions from intact and depolymerized galactomannan. BoGal36A efficiently releases (> 90%) galactose from guar and locust bean galactomannans, resulting in precipitation of the polysaccharides. As compared to other GH36 structures, the BoGal36A 3D model displays a loop deletion, resulting in a wider active site cleft which likely can accommodate a galactose‐substituted polymannose backbone.

## Abbreviations

**CAZy database**, carbohydrate active enzyme database

**DLS**, dynamic light scattering

**GGM**, galactoglucomannan

**GH**, glycoside hydrolase

**G_2_M_5_**, 6^3^, 6^4^‐α‐d‐galactosyl‐mannopentaose

**GM_2_**, 6^1^‐α‐d‐galactosyl‐mannobiose

**GM_3_**, 6^1^‐α‐d‐galactosyl‐mannotriose

**GMOS**, galactose‐substituted manno‐oligosaccharides

**HPAEC‐PAD**, high‐performance anion exchange chromatography with pulsed amperometric detection

**SEC**, size‐exclusion chromatography

**LBG**, locust bean gum

***p*NP‐α‐gal**, p‐nitrophenyl‐α‐galactopyranoside

**PUL**, polysaccharide utilization locus

**RFOS**, raffinose family oligosaccharides

α‐Galactosidases have been classified in glycoside hydrolase (GH) families GH4, GH27, GH36, GH57, GH97 and GH110 based on their sequence similarity, see the Carbohydrate Active Enzymes database (www.cazy.org) 1. GH27 and GH36 α‐galactosidases belong to clan D and share a common (α/β)_8_ fold 2. These families contain cloned and some structurally characterized α‐galactosidases from various prokaryotic and eukaryotic organisms isolated from soil 3, 4, 5, thermal springs 6, 7 and the mammalian gut 8, 9, 10. GH27 α‐galactosidases are active on both terminal and/or internal galactosidic linkages from polysaccharides like galactomannan 11 and galactosylated oligosaccharides 4, while GH36 α‐galactosidases are mainly active on terminal α‐galactosidic linkages in raffinose family oligosaccharides (RFOS) such as raffinose and melibiose 8, 10, 12. Previous studies suggest that unique structural/sequence motifs and the oligomeric state of the enzymes impart the substrate preferences (terminal versus internal galactose linkages) in GH27 4 and GH36 12 α‐galactosidases. Tetrameric α‐galactosidases from both the families have a narrow active site cleft and preferably hydrolyse only terminal α‐galactosidic linkages present in RFOS 4, 12. Recent phylogenetic analysis of GH36 enzymes clusters these sequences into four distinct subgroups 8. The GH36 subgroup I is by far the largest group of the family and contains mainly tetrameric α‐galactosidases active on terminal α‐galactosidic linkages. The majority of biochemically and structurally characterized subgroup I α‐galactosidases 8 are from gut bacteria, such as *Bifidobacterium* and *Lactobacillus* species, and are involved in RFOS utilization 8, 12, 13, 14, 15.

By bioinformatics analysis, we discovered a putative GH36 α‐galactosidase gene (*Bacova_02091)* encoded by a recently discovered polysaccharide utilization locus (PUL) 16 implicated in β‐mannan utilization by the gut bacterium *Bacteroides ovatus* ATCC 8483. In this study, we cloned the gene and characterized the corresponding α‐galactosidase (BoGal36A). In contrast to GH27 α‐galactosidases, GH36 α‐galactosidases have not previously been shown to have significant activity towards galactosylated polymeric β‐mannans (i.e. being able to hydrolyse galactosyl substitutions attached to internal mannose units) 3, 17. Our analysis revealed that BoGal36A belongs to GH36 subgroup I, but has distinct structural and catalytic features associated with β‐mannan utilization rather than RFOS utilization, which is the case for other subgroup I α‐galactosidases as described above. In addition, we relate this knowledge to other *Bacteroides* species, including *B. fragilis* which recently was proposed to catabolize β‐mannan via a new pathway 18.

## Materials and methods

### Growth and transcriptional analysis of *Bacteroides* species on β‐mannans

The *Bacteroides* strains tested were grown in tryptone‐yeast extract‐glucose (TYG) medium 19 or on brain–heart infusion (BHI; Beckton Dickinson) agar containing 10% horse blood (Colorado Serum Co.). Growth measurements on individual substrates were performed in minimal medium (MM) containing a single carbohydrate at 5 mg·mL^−1^ (w/v) final concentration in a 96‐well format using an automated absorbance reader as previously described 16. Transcriptional activation of the *B. ovatus* ATCC 8483 galactomannan PUL on LBG galactomannan and konjac glucomannan was derived from normalized Affymetrix GeneChip data as described in 16.

### BoGal36A sequence analysis

The gene sequence of *Bacova_02091* (GenBank: EDO12201.1, UniProt ID: A7LW87) was mined from genomic sequence data of *B. ovatus* ATCC 8483 for primer design and cloning. A BLASTP search with the protein sequence BoGal36A was performed on the UniProtKB (http://www.uniprot.org/blast/) and PDB databases. The presence of signal peptide was analysed on the signal P server (http://www.cbs.dtu.dk/services/SignalP/) 20. A basic phylogenetic tree was constructed using all the characterized protein sequences from GH36 displayed in the CAZy database along with BoGal36A using maximum likelihood analysis on mega 6.0 21. Multiple sequence alignments of BoGal36A with structurally characterized α‐galactosidases from GH36 subgroup I was performed with the T‐coffee tool (http://www.ebi.ac.uk/Tools/msa/tcoffee/). The alignment was processed in espript3.0 22 and was presented with secondary structure from the crystal structure of *Geobacillus stearothermophillus* α‐galactosidase (AgaB: PDBID‐4FNQ) 10 as reference.

### Cloning of *Bacova_02091* from *Bacteriodes ovatus* ATCC 8483

The *Bacova_02091* gene encoding BoGal36A was amplified by the PCR from genomic DNA of *B*. *ovatus* ATCC 8483, prepared as described previously 19. Primers were designed to include Nco1 and Xho1 sites for cloning. PCR reaction of 50 μL was set up containing MgCl_2_ (2 mm), DNA (5 ng), dNTPs (250 μm), 0.5 μm primers (forward primer: 5′ATACCATGGCCCAAAATATACATTTGTCAACC and reverse primer: 5′CGTCTCGAGCT TAACCTCTTCCAGATAAAGTA), DMSO (2%) and *Pfu* DNA polymerase (2.5 U). The conditions used for PCR were: first cycle at 95 °C for 5 min followed by 35 cycles at 95 °C for 30 s, 55 °C for 30 s and 72 °C for 2 min, and a final cycle at 72 °C for 5 min. The PCR product was double digested by Nco1 and Xho1 enzymes (Thermo scientific, Göteborg, Sweden) and cloned into these sites of the pET28b+ expression vector to generate a clone pB2091 for expression of BoGal36A protein with C‐terminal His_6_ tag. The positive clones containing pB2091 plasmid were confirmed by sequencing and further transformed into a calcium competent BL21 (DE3) *Escherichia coli* strain for BoGal36A expression.

### BoGal36A expression and characterization

*Escherichia coli* BL21 cells containing the pB2091 plasmid, encoding for the BoGal36A protein, was cultured in Luria–Bertani media at 37 °C. The recombinant protein expression was induced by addition of 1 mm (IPTG at mid exponential phase (OD_600 nm_ ~ 0.7). The cells were harvested after incubation at 37 °C for 4 h. BoGal36A was initially released from the induced BL21 cells by suspending the cells in binding buffer (20 mm Tris–HCl, pH 8.0, 300 mm NaCl, 10 mm imidazole, 1 mm phenyl methyl sulphonyl flouride) and lysing it with glass beads (2‐μm diameter, Biospec) by vortexing for 10 times with intervals of 30 s on ice. The supernatant after centrifugation of the lysate at 25 000 × ***g*** for 20 min was loaded on 2 mL His‐Tag resin pre‐equilibrated with the binding buffer. After overnight binding at 4 °C the column was washed with wash buffer (20 mm Tris–HCl, pH 8.0, 300 mm NaCl, 50 mm imidazole) and BoGal36A was eluted in elution buffer (20 mm Tris–HCl, pH 8.0, 300 mm NaCl, 200 mm imidazole). Pure fractions were pooled and buffer was exchanged to 50 mm citrate buffer pH 6.0. Protein concentration was measured on a nano drop ND 1000 spectrophotometer at 280 nm. Absorbances were correlated with the protein concentrations based on the theoretical extinction coefficient: 144 675 M^−1^·cm^−1^. The theoretical molecular weight and the molar extinction coefficient were obtained from the ProtParam tool (http://web.expasy.org/) using the protein sequence of BoGal36A. The eluted protein fractions were also analysed on SDS PAGE (Techtum, 4–12%).

Size‐exclusion chromatography (SEC) was performed to identify the oligomeric state of BoGal36A. 500 μL of 2 mg·mL^−1^ BoGal36A, was loaded on 16/60 Superdex 200 (GE Healthcare, Uppsala, Sweden) pre‐equilibrated with 50 mm citrate buffer pH 6.0 at a flow rate of 0.5 mL·min^−1^ connected to ÄKTA system (GE healthcare). Around 500 μL of γ‐thyroglobulin 669 kDa, apoferritin 443 kDa and β‐amylase 200 kDa (MWGF1000; Sigma‐Aldrich, Steinheim, Germany), was used as molecular weight standards. Two injections of BoGal36A eluted with identical volumes. The apparent molecular weight of oligomeric BoGal36A was calculated based on the calibration curve obtained by plotting partition coefficient (*K*_av_) versus log molecular weight of standard proteins.

### Substrates

The following were purchased from Sigma‐Aldrich: the artificial substrates p‐nitrophenyl‐α‐galactopyranoside (*p*NP‐α‐gal), p‐nitrophenyl‐α‐glucopyranoside (*p*NP‐α‐gluc), p‐nitrophenyl‐β‐galactopyranoside (*p*NP‐β‐gal), p‐nitrophenyl‐β‐glucopyranoside (*p*NP‐β‐gluc) and p‐nitrophenyl‐β‐arabinopyranoside (*p*NP‐β‐ara). RFOS: raffinose, melibiose and stachyose. Galactomannan polysaccharides (β‐1, 4 linked mannan backbone with α‐1, 6 linked galactose substitutions): locust bean gum and guar gum (galactose:mannose ratio ~ 1 : 4 and ~ 1 : 2 respectively) 11. Galactosylated manno‐oligosaccharides (GMOS) are products from hydrolytic galactomannan depolymerization and were purchased from Megazyme International (Bray, Ireland): 6^1^‐α‐d‐Galactosyl‐mannobiose (GM_2_), 6^1^‐α‐d‐Galactosyl‐mannotriose (GM_3_) and 6^3^, 6^4^‐α‐d‐Galactosyl‐mannopentaose (G_2_M_5_). Galactoglucomannan (GGM) was prepared as described previously 23.

### Activity assay and α‐galactosidase specificity

The standard α‐galactosidase assay was performed using 1 mm *p*NP‐α‐gal and release of *p*‐nitrophenol was measured at 405 nm after incubation with BoGal36A at 37 °C, in 50 mm pH 6.0 sodium citrate buffer. The reaction was stopped after 10 min with 1 M Na_2_CO_3_. Assays using *p*NP‐α‐gluc, *p*NP‐β‐gal, *p*NP‐β‐gluc or *p*NP‐β‐ara were performed under similar conditions. The pH optimum was determined by the standard activity assay using buffers between pH 2.0–9.0. The buffers used were 50 mm glycine‐HCl buffer for pH 2.0–3.0, 50 mm sodium citrate buffer for pH 4.0–5.0, 50 mm citrate‐phosphate buffer for pH 6.0–7.0 and 50 mm Tris–HCl buffer for pH 8.0–9.0. Temperature optimum was also determined by standard activity assay at five different temperatures 4, 30, 37, 50 and 70 °C. Incubations were also done for 24 h at various pH and temperatures to determine the pH and temperature stabilities. Michaelis–Menten kinetics was done with *p*NP‐α‐gal as substrate by continuous assay. A 0.1–5 mm concentration of *p*NP‐α‐gal was incubated with 0.1 mg·mL^−1^ of BoGal36A. The rate of the reaction was calculated by monitoring the release of paranitrophenol at 405 nm for 5 min. *K*_M_ and *k*_cat_ values were obtained by fitting the rate of the reaction and substrate concentration in a Michaelis–Menten equation. All the reactions were done in duplicates.

### Substrate specificity using oligo‐ and polysaccharides

RFOS (raffinose, melibiose, stachyose) and GMOS (GM_2_, GM_3_, G_2_M_5_) were incubated with BoGal36A and analysed for galactose release. Around 500 nm of enzyme was incubated with 5 mm of each oligosaccharide in a total volume of 500 μL for maximum of 12 h. Polysaccharides (0.5% Locust bean gum, guar gum or acetylated GGM) were also incubated with 1 μm of enzyme in a total volume of 500 μL. Aliquots of 150 μL were taken at 1 h, 3 h and 12 h and the samples were analysed for galactose release by high‐performance anion exchange chromatography with pulsed amperometric detection (HPAEC‐PAD) (Dionex, Sunnyvale, CA, USA) on a PA10 column with 1% NaOH isocratic elution 24. Specific activity and extent of galactose released from polysaccharides were calculated based on the galactose release after 1 h and 12 h respectively. All the reactions were done in duplicates.

### *k*_cat_/*K*_m_ analysis on GMOS and RFOS

Fifty micromolar of GMOS (GM_2_, GM_3_ and G_2_M_5_) and RFOS (melibiose, stachyose, raffinose) were incubated with BoGal36A (34.2 nm for GMOS and 85.2 nm for RFOS) in 50 mm sodium citrate buffer pH 6.0 at 37 °C. The total reaction volume was 750 μL and aliquots of 125 μL were taken at 0, 2, 5, 10, 20 and 30 min for each of the substrates and the reaction was stopped by addition of 10 μL 5% NaOH. All the reactions were done in duplicates. The decrease in substrate concentration for each of the GMOS and RFOS was analysed by HPAEC‐PAD on a PA100 column as described previously 25. *k*_cat_/*K*_M_ was calculated according to the Matsui equation 26 by plotting Ln (*S*_o_/*S*_t_) as a function of time (*t*) similar to previous studies 24. *S*_o_ is the initial substrate concentration at time zero, and *S*_t_ is the substrate concentration at time (*t*).

### DLS analysis and guar gum aggregation

The ability of BoGal36A to modify the size of guar gum aggregates was followed by dynamic light scattering (DLS) experiments. Around 500 μL reaction volume was set up with 0.25% guar gum in 50 mm citrate buffer pH 6.0 and 0.1 mg·mL^−1^ of BoGal36A enzyme. The reaction was carried out in a microvolume quartz cuvette at 37 °C in a zeta sizer‐Nano ZS90 (Malvern Instruments, Worcestershire, UK). The change in particle size in the reaction mixture was followed every 10 min for 16 h. The particle size of the polysaccharide was measured as *Z*_ave_ mean on zeta sizer (Nano ZS90), based on the absorption at 488 nm. *Z*_ave_ mean is the mean size diameter calculated by considering all the other factors like viscosity, Boltzmann constant and diffusion 27, 28. It more accurately represents the particle size during the course of reaction. Aliquots of 20 μL were taken at four different time points: 0, 4, 12 and 16 h to analyse the galactose release at different time intervals on HPAEC‐PAD PA10 column. The % galactose released was calculated based on the galactose: mannose ratio of 0.7 : 1 in guar gum.

### Homology model

A homology model of BoGal36A was built in Swiss PDB model work space (http://swissmodel.expasy.org/) using the crystal structure (chain A) of a α‐galactosidase from *Geobacillus stearothermophillus* AgaA (PDBID ‐ 4FNP) 10, with 34% sequence identity as template. The quality of the 3D model was assessed based on Q mean score and gave a *Z* value of – 0.576. Ramachandran plot analysis showed 0.5% of amino acid residues in disallowed regions, 97% of residues in the allowed region and 1.5% in generously allowed regions. All the figures were drawn in pymol (Molecular Graphics 122 System, Version 1.5.0.4 Schrödinger, and LLC).

## Results

**Figure 1:**
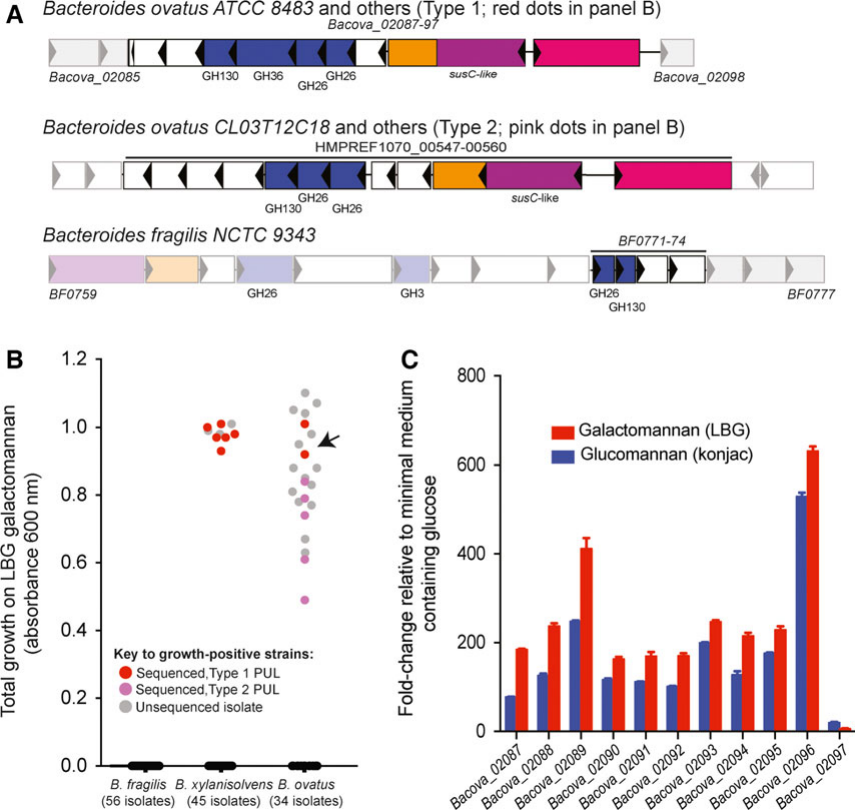
*Bacteroides* genes involved in galactomannan degradation and growth of selected species on β‐mannans. (A) Gene clusters from *B. ovatus* (Type 1) and *B. fragilis* (bottom) that have previously been implicated in β‐mannan degradation 16, 18. Type 2 putative β‐mannan PUL was discovered in this study. Surrounding genes that were not shown to be transcriptionally active in response to β‐mannan (for *B. ovatus*)/or were not tested for functions in β‐mannan degradation are showed as partially transparent. Note that flanking *BF0771‐74* there is a GH26‐containing polysaccharide utilization locus. (B) Growth of strains from three different *Bacteroides* species on LBG galactomannan. Growth ability of the strains with Type 1 PUL is highlighted in red (*B. ovatus* ATCC *8483* is indicated by an arrow), strains with Type 2 PUL are marked in pink. Sequenced *B. ovatus* and *B. xylanisolvens* strains with no growth on galactomannan do not contain either of the PULs. (C) Transcriptional activation of the *B. ovatus* ATCC *8483 Bacova_02087‐97,* Type 1 β‐mannan PUL on konjac glucomannan and LBG galactomannan. More information related to strains numbers and the homologous/partially homologous PULs is presented in supplementary material (Table S1, Fig. S1).

The genetic loci proposed to be involved in β‐mannan utilization in *B. ovatus* ATCC8483 (Type 1, Fig. 1A) and *B. fragilis* NCTC 9343, respectively, are shown in Fig. 1A. It can be concluded that the loci lack overall homology, one of the differences being absence of a possible α‐galactosidase gene for *B. fragilis*. On the other hand, we found that *B. xylanisolvens D 22* has a genetic locus (HMPREF0106_00419 to HMPREF0106_00429) with overall homology to the type 1 locus of *B. ovatus*. Growth studies on galactomannan substrate shows that at least some of the tested *B. ovatus* and *B. xylanisolvens* strains efficiently utilize galactomannan as a substrate, while none of the tested *B. fragilis* species were able to grow on galactomannan (Fig. 1B), including the type strain *B. fragilis* NCTC 9343. Comparative genomic analysis of the sequenced *B. ovatus* and *B. xylanisolvens* strains with high growth on galactomannan (Fig. 1B) showed the presence of a genetic locus homologous to type 1 β‐mannan PUL of *B. ovatus* ATCC 8483 (Figs 1A and S1). Transcriptional activation analysis for growth of *B. ovatus* ATCC 8483 on galactomannan showed upregulation of the type 1 β‐mannan utilization locus (gene cluster *Bacova_02087‐97),* (Fig. 1C). This gene cluster contains the GH36 α‐galactosidase gene, locus tag *Bacova_02091,* along with two putative GH26 β‐mannanase genes (locus tags *Bacova_02092* and *Bacova_02093*) and a putative GH130 gluco‐mannophosphorylase (locus tag *Bacova_02090*). Some of the *B. ovatus* strains with positive growth on galactomannan lack the type 1 β‐mannan PUL, but have a partially homologous PUL (Type 2), which, however, lacks a α‐galactosidase (Figs 1A,B and S1). α‐Galactosidase activity is needed for hydrolysis of galactosyl substitutions present in galactomannans, albeit this is a function known for GH27 but not for GH36 α‐galactosidases, as explained above. The presence of a GH36 α‐galactosidase gene in a β‐mannan PUL motivated us to clone and study the properties of the recombinant enzyme BoGal36A.

### Sequence analysis of BoGal36A

**Figure 2:**
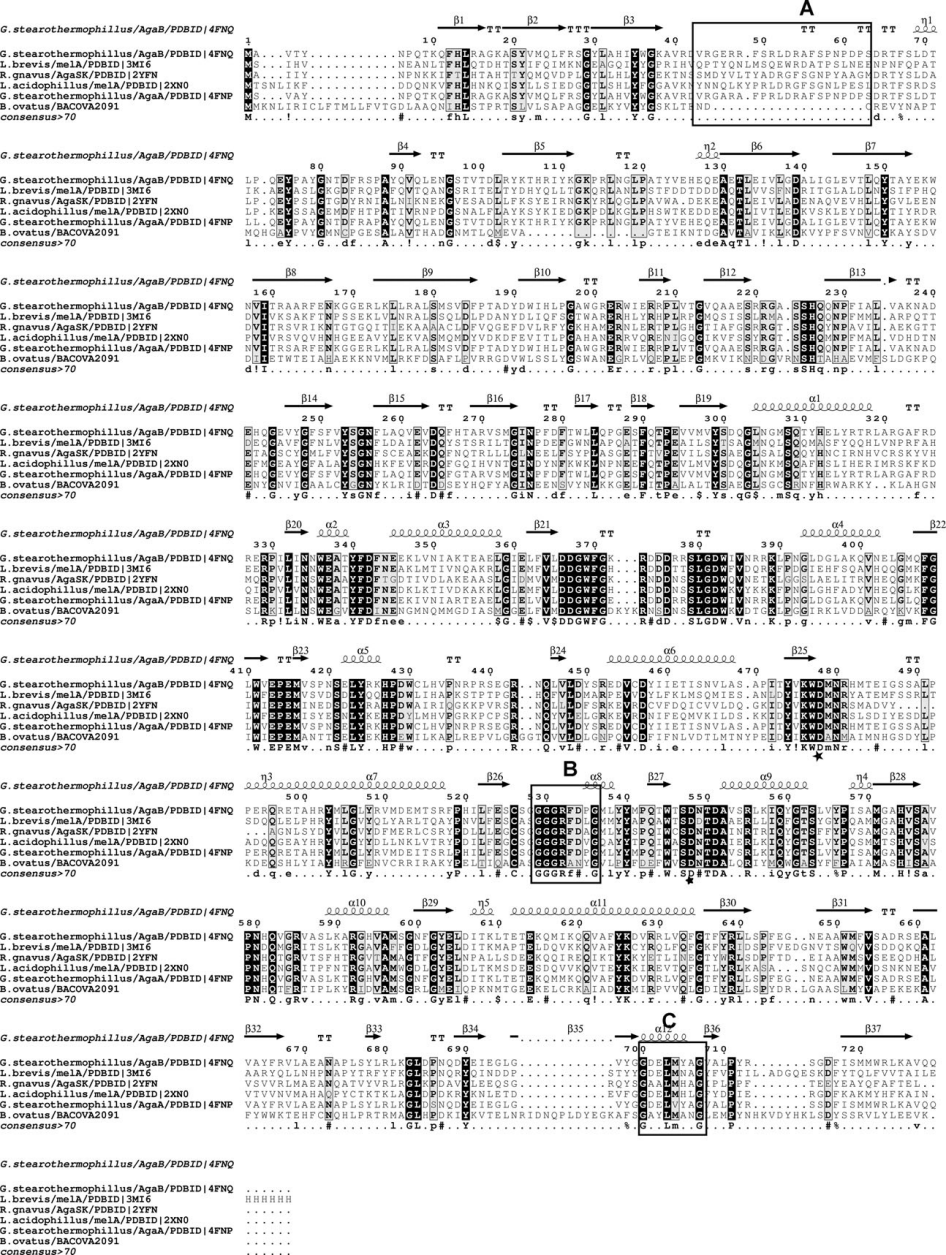
Multiple alignment of structurally characterized GH36 α‐galactosidases from Subgroup I. The subgroup classification is based on Fredslund *et al*. 12 and secondary structure elements from GH36 α‐galactosidase from *G. stearothermophillus*: PDB ID 4FNQ. Completely conserved residues are marked black and partially conserved residues are marked as grey. Catalytic amino acids: nucleophile D479 and acid/base D549 are marked. Unique features: (A) missing loop in BoGal36A (B) CXXGXXR motif involved in galactose recognition in subgroup1 and (C) GXXLXXXG motif involved in tetramer formation in subgroup I.

BoGal36A has an N‐terminal signal peptide but no lipid anchor attachment. The protein is thus predicted to be secretory and soluble. Its potential presence in periplasm or extracellular environment is, however, difficult to predict. A BlastP search using the sequence of BoGal36A as a query resulted in many putative α‐galactosidases from *Bacteroides* species. The highest identity (36%) for a characterized α‐galactosidase was that from a symbiotic bacterium *Flavobacteria sp*. TN17 isolated from the gut of the wood‐feeding insect *Batocera horsefeldi* 29. The phylogenetic analysis clusters BoGal36A to subgroup I 12 of GH36, which contains the majority of structurally and biochemically characterized α‐galactosidases from raffinose utilization loci of other gut bacteria (Fig. S2). The sequence alignment with structurally characterized GH36 α‐galactosidases from subgroup I indicate conserved amino acids involved in galactose recognition and a GXXLXXXG motif unique for α‐galactosidases from subgroup I (Fig. 2) proposed to be involved in protein tetramerization 12.

### Cloning and basic characterization

The gene sequence encoding BoGal36A without the predicted secretion signal was amplified from genomic DNA of *B. ovatus* ATCC 8483 and cloned into the pET28b+ vector with a C‐terminal His_6_ tag. The overexpressed protein was purified by His‐Tag purification. A single band corresponding to ~ 81 kDa, observed on SDS PAGE (Fig. S3) was similar to the theoretical molecular weight of BoGal36A (80.7 kDa). In SEC analysis, BoGal36A elutes as a single peak at 61.3 mL (Fig. S4) which corresponds to 290 kDa, slightly below the theoretical molecular weight of a tetramer (324 kDa).

**Figure 3:**
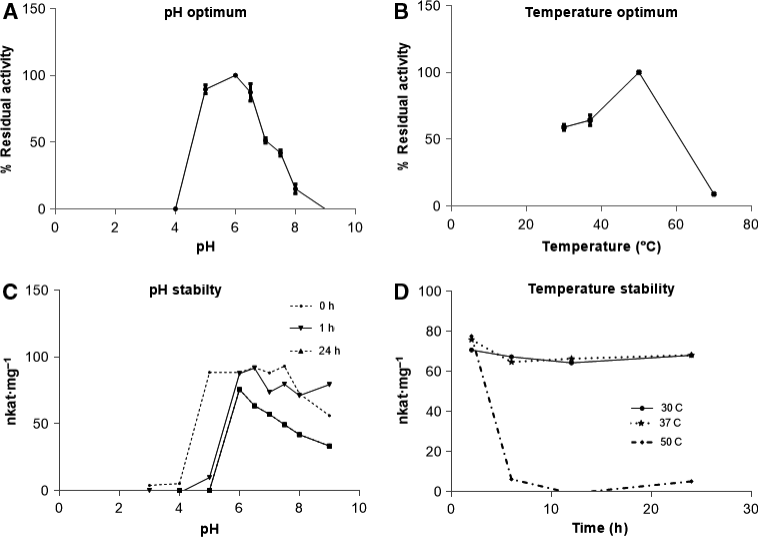
Effect of pH and temperature on activity of BoGal36A: (A) pH optimum: hydrolysis of *p*NP‐ α‐gal for 10 min at pH 2–9. (B) Temperature optimum: hydrolysis of *p*NP‐ α‐gal at pH 6 for 10 min. (C) pH stability: activity dependence from pH 3–9 for 24 h at 37 °C (D) Temperature stability: activity dependence at pH 6 for 24 h at 30, 37 , 50 °C.

Recombinant BoGal36A is active on *p*NP‐α‐gal, but not on the other tested *p*NP‐glycosides. Thus, basic characterization was done with *p*NP‐α‐gal as substrate. BoGal36A has pH optimum of 6.0 and optimum temperature of 50 °C (Fig. 3B) over an assay time of 10 min but is not stable if the incubation is prolonged. However, the enzyme retains greater than 85% activity at pH 6.0 (Fig. 3C) when incubated at 37 °C for 24 h. Thus, the optimum conditions of pH 6.0 and 37 °C were used in further incubations for analysis of kinetic parameters and natural substrate specificity. BoGal36A has *K*_m_ of 0.132 ± 0.02 mm and *k*_cat_ of 4774 ± 202 min^−1^ using *p*NP‐α‐gal as substrate.

### Substrate specificity on galactose containing substrates

**Figure 4:**
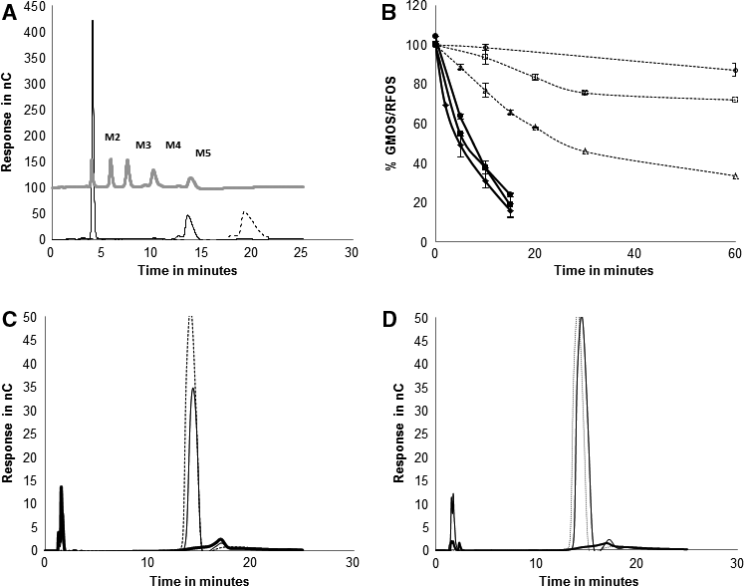
Galactose release analysis: (A) G_2_M_5_ hydrolysed to M_5_, analysed on PA100 column. (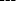) indicates G_2_M_5_ at 0 h. Mannose standards M2–M5 and galactose peak is also indicated. (B) Degradation curves for galactose‐substituted oligosaccharides. (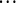) indicates RFOS (—) indicates GMOS. Markers indicate (●) GM_2_, (♦) GM_3_, (■) G_2_M_5_, (▵) melibiose (□) Raffinose and (○) stachyose. Galactose release analysed on PA10 column for BoGal36A hydrolysed guar gum (C) and locust bean gum (D). Grey indicates 1 mm galactose standard. Black line indicates galactose release from polysaccharides and bold black line indicates sample at 0 h.

The specific activities for the galactomannans guar gum and LBG and for GGM are 64.2 ± 3.7 min^−1^, 70.2 ± 6.96 min^−1^ and 9.6 ± 0.3 min^−1^ respectively. BoGal36A had a higher specific activity of 1260 ± 32 min^−1^ on G_2_M_5_. Both the galactose residues were released from G_2_M_5_ (Fig. 4A) and a single galactose group was released from GM_2_ and GM_3_. Galactose was also released from RFOS (melibiose, raffinose, stachyose), but less efficiently than from GM_2_, GM_3_ and G_2_M_5_ as shown by substrate depletion curves (Fig. 4B). About 90% of galactose was released from LBG and guar gum galactomannan after incubating 0.5% of polysaccharides (Fig. 4C,D). Precipitation of guar galactomannan was observed due to aggregation of polysaccharide backbone after galactose removal. Only 10% galactose was removed from GGM.

### *k*_cat_/*K*_m_ analysis: Terminal versus internal galactose specificity

BoGal36A hydrolyses internally linked α‐1, 6 galactose residues from the GMOS: GM_2_, GM_3,_ and G_2_M_5_ with 13 times higher catalytic efficiency (*k*_cat_/*K*_m_) compared to terminal α‐galactose residues from the RFOS raffinose, stachyose and melibiose (Fig. 4B and Table 1). It has similar catalytic efficiency for GM_2_, GM_3_ and G_2_M_5_, indicating that the length of the mannan backbone or the frequency of galactose substitutions does not affect the catalytic efficiency of the enzyme. In contrast, for RFOS the catalytic efficiency decreases for raffinose and stachyose compared to melibiose (Table 1). Based on *k*_cat_/*K*_m_ analysis it can be ventured that BoGal36A can accommodate mannosyl‐substituted galactose (GMOS) in the active site better than galactosylated oligosaccharides from the RFOS family.

**Table 1:** Kinetic properties of BoGal36A on different galactose substrates

Substrate	Structure	*k*_cat_/*K*_m_ (M^−1^ Min^−1^)
pNp‐α‐gal		3.6 × 10^7^ ± 8.9 × 10^5^
RFOS		
Raffinose	αGal‐(1,6)‐αGlc^a^	2.6 × 10^5^ ± 2.2 × 10^4^
Melibiose	αGal(1,6)‐αGlc‐(1,2)‐βFru	5.16 × 10^4^ ± 1.7 × 10^3^
Stachyose	αGal(1,6)‐αGal(1,6)‐aGlc‐(1,2)‐βFru	3.16 × 10^4^ ± 2.2 × 10^3^
GMOS		
GM_2_	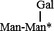	3.5 × 10^6^ ± 1.0 × 10^5^
GM_3_	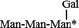	3.24 × 10^6^ ± 1.7 × 10^5^
G_2_M_5_	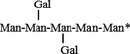	3.15 × 10^6^ ± 2.3 × 10^5^

a Represents reducing end of the oligosaccharide

### DLS experiments for guar gum aggregation

**Figure 5:**
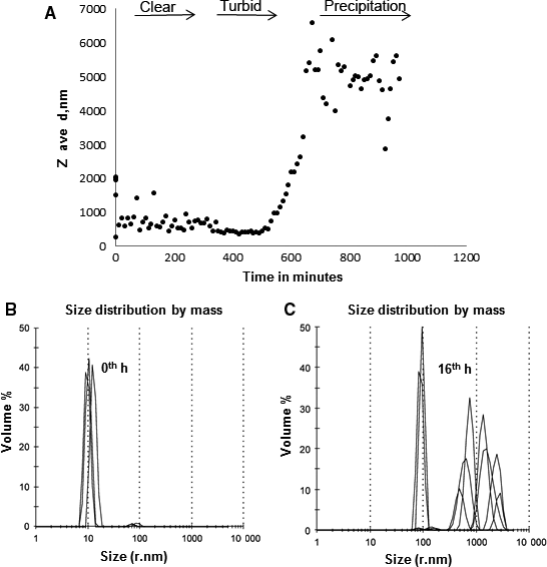
DLS analysis of guar gum galactomannan: (A) mean particle size distribution (*Z*_ave_) of guar gum with galactose removal by BoGal36A over time for 16 h. The real‐time change in particle size is shown for 0 h (B) and 16 h (C). In initial time points at 4 h, where the galactose removal is less than 40%, the change in particle size is not significant. The particle size increases to ~ 1 μm after 16 h when the extent of galactose release reached 90%.

Addition of BoGal36A actively removes the galactosyl residues from guar gum galactomannan, thus promoting aggregation of mannan backbone. Initially, the guar gum galactomannan has a particle size diameter between 10 and 100 nm (Fig. 5B). Removal of galactose substitutions resulted in aggregation and the increase in particle size to 1 μm is proportional to the extent of galactose removal (Fig. 5). The graph plotted (Fig. 5A) shows the change in particle size of guar gum galactomannan displayed as *Z*_ave_ mean versus time during the course of galactose removal by BoGal36A. The real‐time change in particle size is shown in Fig. 5B,C for 0 and 16 h respectively. At 4 h, where the galactose removal was less than 40%, the change in particle size was not significant. The galactose removal reached 65% at 8 h and there was a steady increase in *Z*_ave_. The particle size increased to ~ 1 μm after 16 h when the extent of galactose release reached 90%.

### Homology model

**Figure 6:**
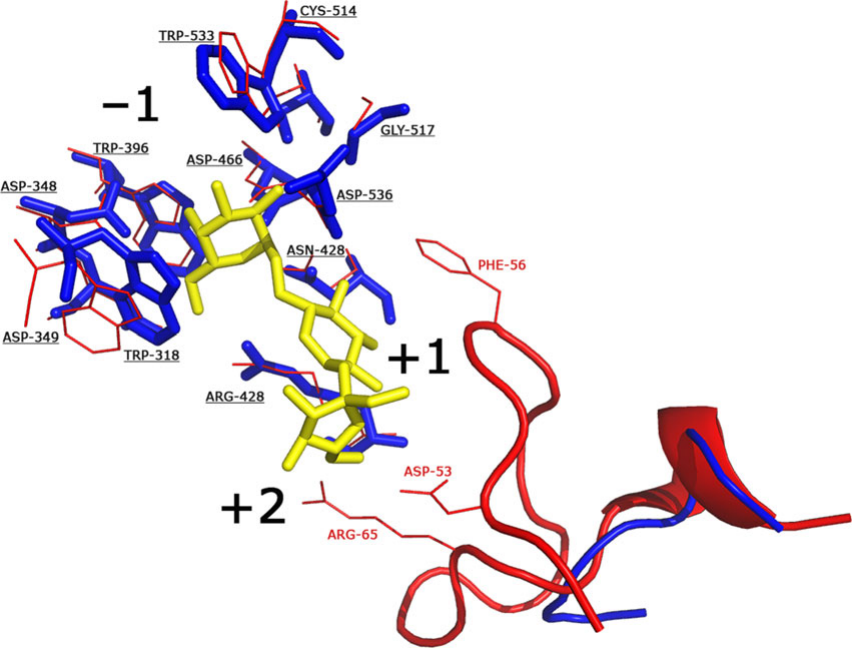
Close up view of BoGal36A overlay on AgaA with raffinose bound in the active site pocket 10: Amino acids from the template structure AgaA (red), with raffinose (yellow) in the active site, and the BoGal36A model (blue). Phe 56, Asp 53 and Arg 65 (labelled red, AgaA numbering) are part of the loop that is lacking in BoGal36A. The positive and negative subsites are marked as −1, +1 and +2 respectively. Amino acids involved in galactose recognition at the −1 subsite are conserved and are underlined (BoGal36A numbering).

The observed differences in BoGal36A (not optimal RFOS activity, rather active on galactomannan and GMOS) and other gut bacterial GH36 α‐galactosidases (involved in RFOS hydrolysis) led us to try to find structural differences. The BoGal36A 3D model was based on the template structure of AgaA from *G. stearothermophillus*, which is tetrameric in solution 10. The tetrameric BoGal36A model was generated assuming that the orientation of the individual monomers in the modelled BoGal36A is similar to that of AgaA. Comparison using an active site overlay of modelled BoGal36A showing raffinose bound in the active site of AgaA (PDB: FN0) indicates a conserved −1 subsite involved in galactose recognition as compared to the template (Fig. 6).

The main differences are seen in the +1 and +2 subsites of AgaA. The P‐related loop (AgaA amino acids 55–66) of the AgaA structure is absent in BoGal36A (Fig. 6). This loop provides stacking and hydrogen‐bonding substrate interactions (to Glu and Frc moieties of raffinose) via residues in the +1 and +2 subsites (AgaA amino acids Phe 56, Arg65, Asp53) 10. Furthermore, we made a structural overlay of all structurally characterized GH36 subgroup I α‐galactosidases (the same as used Fig. 2) which are preferentially active on terminal α‐galactosidic linkages. The P‐loop is spatially conserved in all these α‐galactosidases (i.e. except BoGal36A) and restricts the space in the positive subsites of the active site cleft. The absence of the loop in BoGal36A is likely to provide additional space for a polymannose backbone and/or allow accommodation of galactose substitutions.

## Discussion

Gene clusters implicated in β‐mannan utilization have been suggested for some *Bacteriodes* species and a few other bacteria which occur in the human gut 16, 18, 30, 31. However, only limited data are available on the functional proteins involved in the utilization of galactomannan as a carbon source in such bacteria 32, 33, 34, 35. *B. ovatus* has previously been shown to utilize galactomannan as a carbon source 16, 33. α‐Galactosidases have been characterized from *B. ovatus* grown on galactomannan 32, 33. However, no genomic data or sequence data are available relating the activity to protein sequences, GH family or genetic locus. In the current study, we show that the *B. ovatus* β‐mannan PUL 16 includes a gene for a GH36 α‐galactosidase (BoGal36A) transcriptionally upregulated, along with the PUL, during growth on galactomannan (Fig. 1C). The genetic coregulation and the biochemical characterization of BoGal36A suggest a new role for a GH36 α‐galactosidase, that is, in galactomannan degradation, a function rather observed for GH27 enzymes from bacteria and fungi 3, 36.

BoGal36A belongs to GH36 α‐galactosidases subgroup I, hitherto suggested to contain enzymes that mainly have evolved to hydrolyse RFOS substrates with a narrow active site formed by enzyme tetramers 10, 12. Interestingly, while being tetrameric, BoGal36A is more efficient in removing internal galactosidic linkages from GMOS of DP 2‐5, compared to RFOS (Table 1), and also releases 90% galactose from guar gum and LBG galactomannans, which is not shown for any other characterized GH36 α‐galactosidase. Previously characterized, but unidentified, α‐galactosidases from *B. ovatus* cannot hydrolyse galactose residues from intact galactomannans 32, 37. An overlay of the modelled tetrameric BoGal36A active site with that of the active site of the RFOS‐hydrolysing AgaA α‐galactosidase reveals likely architectural differences between BoGal36A and other subgroup I GH36 enzymes. The absence of a loop in the N‐terminal region (residues 50–66, AgaA numbering) of BoGal36A, containing aromatic residues involved in stacking interactions in the positive subsites of AgaA, can likely provide the additional space to accommodate galactose substitution carried by a polymannose backbone (Fig. 6).

As it appears, BoGal36A has evolved to hydrolyse internal galactosyl decorations from GMOS and/or galactomannans, an activity previously known for GH27 α‐galactosidases that act synergistically with β‐mannanases for effective galactomannan utilization 11, 38. The β‐mannan PUL upregulated in the presence of galactomannan (Fig. 1C) also encodes two putative GH26 β‐mannanases along with BoGal36A (Fig. 1A). Known β‐mannanases hydrolysing galactomannans by endo‐action are often restricted by galactosyl substitution present on the β‐mannan chain 2, 39, 40. The transcriptional regulator (*Bacova_02097*) of the *B. ovatus* β‐mannan PUL, is also sensitive to galactosyl substitutions, and cannot bind di‐galactosyl mannopentaose (G_2_M_5_) but can bind undecorated β‐mannan oligosaccharides 16. In line with these observations, it is likely that the function of BoGal36A is removal of internal galactose residues from galactomannans and/or GMOS produced by the putative β‐mannanases, enabling the effective utilization of galactomannan as the carbon source. Additionally, BoGal36A can also be effectively utilized as a biotechnological tool for modifying the properties of galactomannans (Fig. 5).

Exo‐glycosidases, such as BoGal36A, described in this study may play an essential role in the *Bacteroidetes* ability to utilize several heteroglycans, acting together with endohydrolases that depolymerize glycan backbones. As an example, exoglycosidases belonging to GH31 (α‐xylosidases) and GH2 (β‐galactosidases) were shown to play an important role in xyloglucan utilization of *B. ovatus* 19. Recently, α‐mannan utilization in *B. thetaiotamicron* was described and also involves exo‐acting GHs such as a α‐1, 6 mannosidase acting together with endo‐acting GHs to effectively utilize the highly branched yeast α‐mannan as carbon source 41.

## Conclusion

This study gives insight into the GHs involved in the β‐mannan utilization of *Bacteroides* species, with focus on the potential role of a GH36 α‐galactosidase, evolved to hydrolyse the internal galactose residues of galactomannan substrates. Based on the genomic context and the substrate preferences of BoGal36A it can be hypothesized that BoGal36A act in cooperation with the predicted GH26 β‐mannanase(s) of the same PUL for effective galactomannan utilization. The competitive environment in the gut may be a contributing factor to the evolution of the structural‐functional difference of BoGal36A compared to the characterized homologs in subgroup I of GH36. Furthermore, the study also exemplifies that the human gut microbiome can be mined for novel enzymes, e.g, for certain applications; in this case for the degalactosylation of galactomannans.

## Author contributions

HS, NK and EM defined the overall research topic. HS, NK, EM and SKR planned the study. SKR, NAP, VB and HB conducted experiments. All authors interpreted the data. SKR, HS, VB, NK and EM wrote the MS.

## Supporting information


**Table S1.** *Bacteroides ovatus* and *B. xylanisolvens* strains with known genomic sequence data.
**Fig. S1.** Comparative genomic view of the predicted β‐mannan utilisation loci homologous to the Type 1 and Type 2 PULs shown in Fig. 1A, present in *B. ovatus* and *B. xylanisolvens* strains that showed positive growth on galactomannan (Fig. 1B).
**Fig. S2.** Phylogenetic analysis including BoGal36A and GH36 enzymes listed as characterised in the CAZy database (characterization on gene or protein‐level).
**Fig. S3.** SDS PAGE of BoGal36A: Lane 1 and 2 represents purified BoGal36a after His tag purification from two different batches.
**Fig. S4.** SEC analysis of BoGal36A.
