# Supplementary material for: A β‐mannan utilization locus in Bacteroides ovatus involves a GH36 α‐galactosidase active on galactomannans
Source: FEBS Lett. 2016 Jun 28;590(14):2106–18. doi: 10.1002/1873-3468.12250 (PMC5094572; doi:10.1002/1873-3468.12250)
Supplement: Supplementary file 1 — Table S1. Bacteroides ovatus and B. xylanisolvens strains with known genomic sequence data. Fig. S1. Comparative genomic view of the predicted β‐mannan utilisation loci homologous to the Type 1 and Type 2 PULs shown in Fig. 1A, present in B. ovatus and B. xylanisolvens strains that showed positive growth on galactomannan (Fig. 1B). Fig. S2. Phylogenetic analysis including BoGal36A and GH36 enzymes listed as characterised in the CAZy database (characterization on gene or protein‐level). Fig. S3. SDS PAGE of BoGal36A: Lane 1 and 2 represents purified BoGal36a after His tag purification from two different batches. Fig. S4. SEC analysis of BoGal36A. [file FEB2-590-2106-s001.docx]

**Supplementary material**

**Table S1**: *B. ovatus* and *B. xylanisolvens* strains with known genomic sequence data.

| **Organism** | **Strain** | **Type 1**  **β-mannan PUL*** | **Type2**  **β-mannan PUL^#^** | **Growth on galactomannan** |
| --- | --- | --- | --- | --- |
| *B. ovatus* | ATCC 8483^1^  NLAE-zl-C34^2^  NLAE-zl-C11^2^  CL02T12C04^1^  3_8_47FAA^1^  CL03T12C18^1^  NLAE-zl-H304^2^  NLAE-zl-H361^2^  3_1_23^1^  D2 (2_1_39) ^1^  NLAE-zl-H59^2^  NLAE-zl-H73^2^ | Yes  No  No  Yes  No  No  No  No  No  No  No  No | No  No  No  No  No  Yes  No  No  Yes  Yes  Yes  Yes | ++  -  -  ++  -  +  -  -  +  +  +  + |
| *B. xylanisolvens* | NLAE-zl-C29^2^  NLAE-zl-G310^2^  NLAE-zl-G421^2^  NLAE-zl-C182^2^  NLAE-zl-C339^2^  2_1_22^1^  3_1_13^3^  D1 (1_1_22) ^1^  NLAE-zl-P393^2^  NLAE-zl-P352^2^  NLAE-zl-P727^2^  NLAE-zl-P736^2^  D22 (1_2_8) ^2^  NLAE-zl-P732^1^  1_1_30^2^  2_2_4^1^  CL03T12C04^1^  NLAE-zl-H194^2^ | No  No  No  No  No  No  No  No  Yes  Yes  Yes  Yes  Yes  Yes  No  No  No  No | No  No  No  No  No  No  No  No  No  No  No  No  No  No  No  No  No  No | -  -  -  -  -  -  -  -  ++  ++  ++  ++  ++  ++  -  -  -  - |

‘++’O.D > 0.9; ‘+’, O.D < 0.9; ‘-’ no growth

* Type 1 PUL homologous to *B. ovatus* ATCC8483, shown in fig1A

# Type 2 PUL homologous to *B. ovatus* CL03T12C18, shown in fig1A

1 genome sequence from <http://www.ncbi.nlm.nih.gov/>

2 genome sequence from <https://img.jgi.doe.gov/>

3genomic sequence from <https://www.broadinstitute.org/>

#

**Fig S1. Comparative genomic view of homologous β-mannan PULs**

**
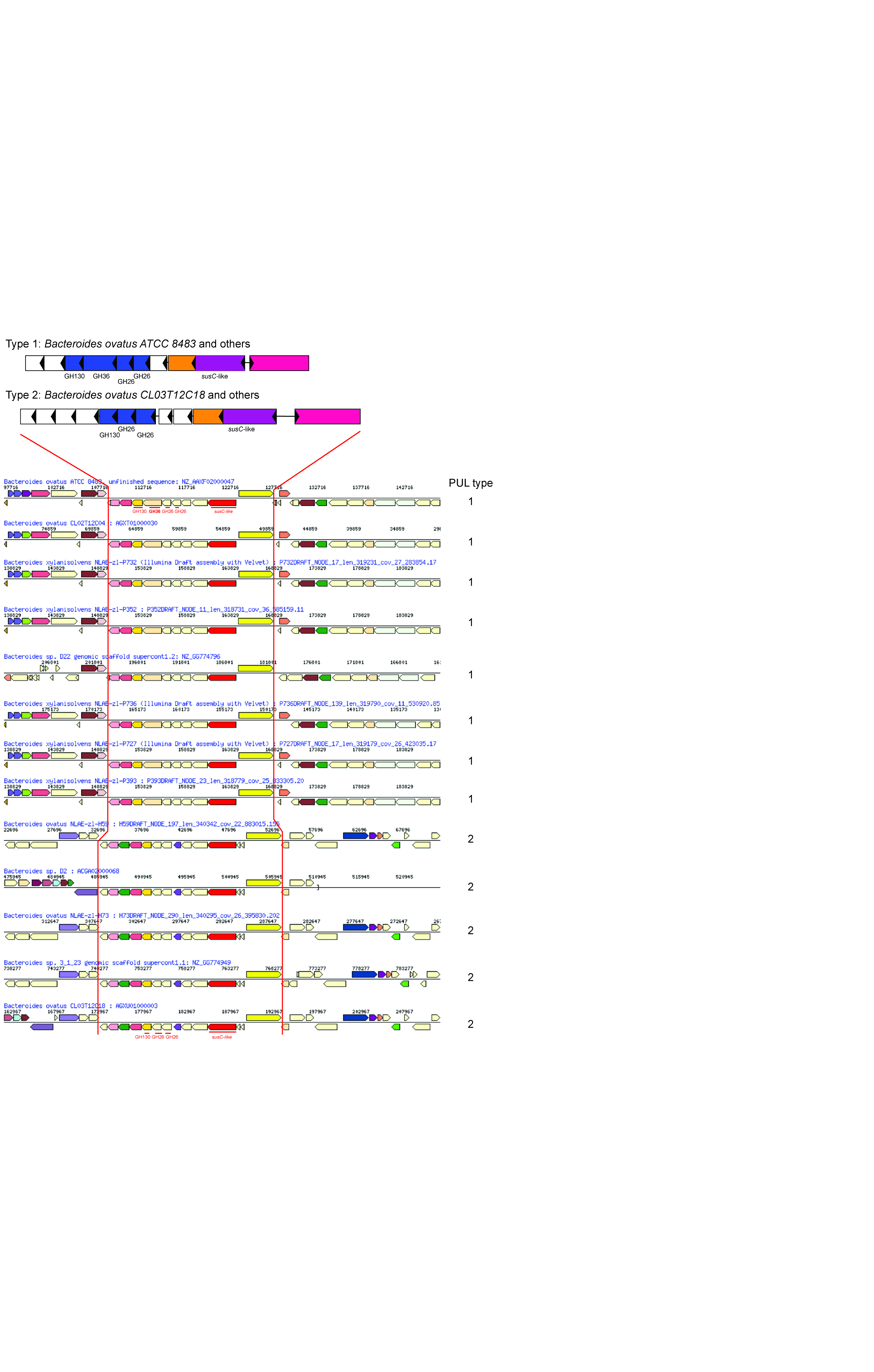
**

**Fig S1**: Comparative genomic view of the predicted β-mannan utilisation loci homologous to the Type1 and Type 2 PULs shown in Fig 1A, present in *B. ovatus* and *B. xylanisolvens* strains that showed positive growth on galactomannan (Fig 1B). The genes encoding GHs and *susC* –like proteins are marked in red on the first and the last sequence respectively. Note that the type 2 PUL lacks the GH36 gene. The figure is based on the output from a comparison done with *susC*-like gene as the search homolog (<https://img.jgi.doe.gov/> ).

**Fig S2. Phylogenetic analysis**

**Fig S2:** Phylogenetic analysis including BoGal36A and GH36 enzymes listed as characterised in the CAZy database (characterization on gene or protein-level). The subgroup division is according to Fredslund et al. [10].

**Fig S3. SDS PAGE OF BoGal36A**

70kda


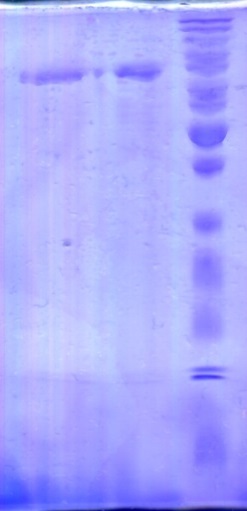


85kda

1 2 3

60kda

50kda

100kda

**Fig S3:** SDS PAGE of BoGal36A: Lane 1 and 2 represents purified BoGal36a after His tag purification from two different batches. Lane 3 shows the protein ladder.

**Fig S4**. **SEC analysis of BoGal36A**


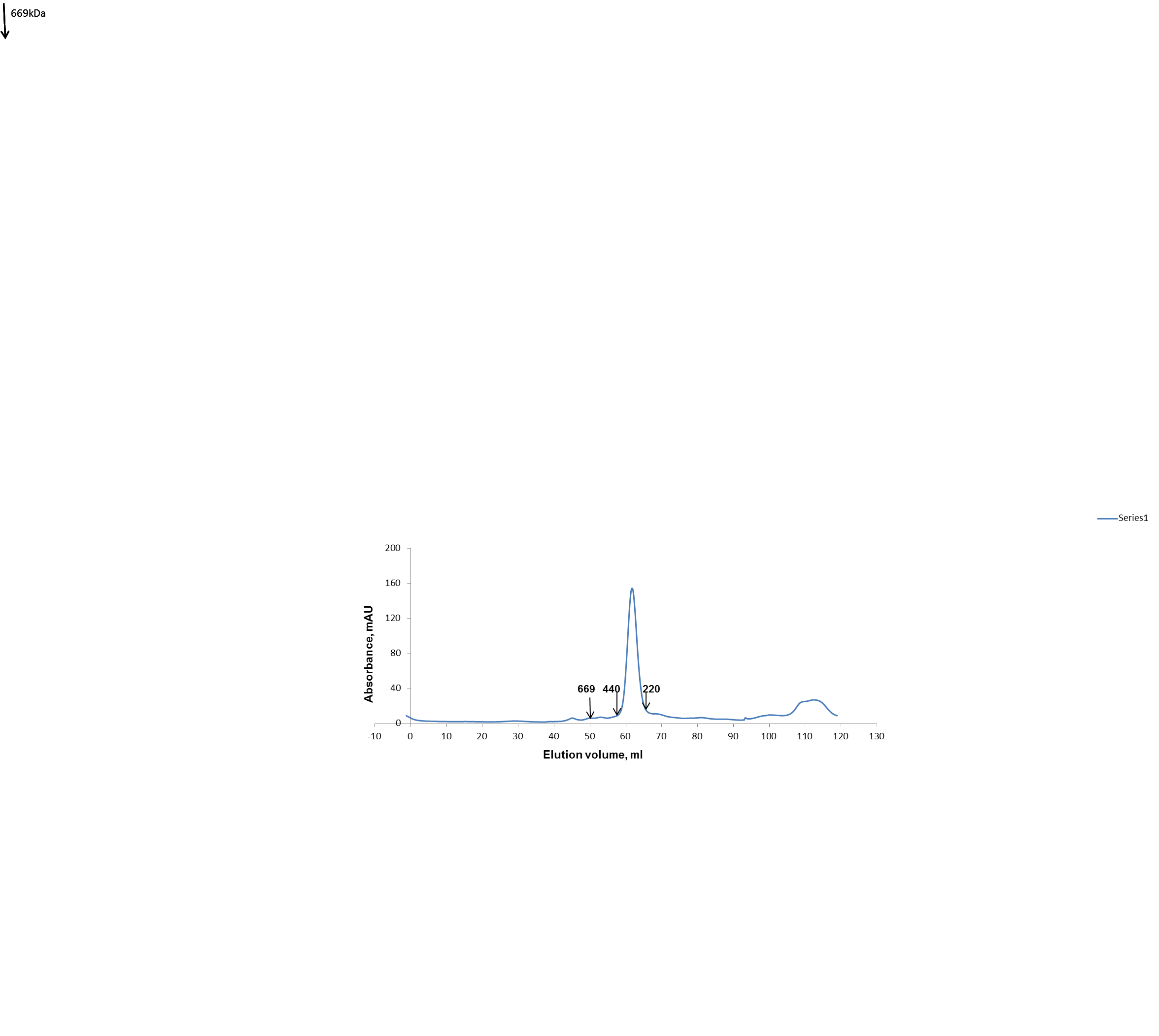


**Fig S4**: SEC analysis of BoGal36A. The elution volume of calibration proteins are marked with corresponding molecular weights in kDa. Thyroglobulin 660kDa, Apoferritin 440kDa, Amylase 200kDa
